# Identifying temporal variations in burn admissions

**DOI:** 10.1371/journal.pone.0286154

**Published:** 2023-06-08

**Authors:** Robel T. Beyene, David P. Stonko, Stephen P. Gondek, Jonathan J. Morrison, Bradley M. Dennis

**Affiliations:** 1 Division of Acute Care Surgery, Department of Surgery, Vanderbilt University Medical Center, Nashville, Tennessee, United States of America; 2 Department of Surgery, The Johns Hopkins Hospital, Baltimore, Maryland, United States of America; 3 Mayo Clinic Division of Vascular and Endovascular Surgery, Rochester, MN, United States of America; Jeonbuk National University, REPUBLIC OF KOREA

## Abstract

**Background:**

Variations in admission patterns have been previously identified in non-elective surgical services, but minimal data on the subject exists with respect to burn admissions. Improved understanding of the temporal pattern of burn admissions could inform resource utilization and clinical staffing. We hypothesize that burn admissions have a predictable temporal distribution with regard to the time of day, day of week, and season of year in which they present.

**Study design:**

A retrospective, cohort observational study of a single burn center from 7/1/2016 to 3/31/2021 was performed on all admissions to the burn surgery service. Demographics, burn characteristics, and temporal data of burn admissions were collected. Bivariate absolute and relative frequency data was captured and plotted for all patients who met inclusion criteria. Heat-maps were created to visually represent the relative admission frequency by time of day and day of week. Frequency analysis grouped by total body surface area against time of day and relative encounters against day of year was performed.

**Results:**

2213 burn patient encounters were analyzed, averaging 1.28 burns per day. The nadir of burn admissions was from 07:00 and 08:00, with progressive increase in the rate of admissions over the day. Admissions peaked in the 15:00 hour and then plateaued until midnight (p<0.001). There was no association between day of week in the burn admission distribution (p>0.05), though weekend admissions skewed slightly later (p = 0.025). No annual, cyclical trend in burn admissions was identified, suggesting that there is no predictable seasonality to burn admissions, though individual holidays were not assessed.

**Conclusion:**

Temporal variations in burn admissions exist, including a peak admission window late in the day. Furthermore, we did not find a predictable annual pattern to use in guiding staffing and resource allocation. This differs from findings in trauma, which identified admission peaks on the weekends and an annual cycle that peaks in spring and summer.

## Introduction

Significant research exists on the subject of resource utilization in burns, but the majority of it has been focused on resources used over the length of admission and beyond [1–6] or disaster management [7–13], rather than predicting acute needs at admission. Understanding variations in burn admissions would be beneficial in allocating staff and resource availability within a burn center, and across the burn system. If peak admission times were regular and predictable based on historic distributions, staffing could be altered to meet those needs and resources could be appropriately marshalled. Time of day, day of week, and annual variations are anecdotally discussed at the institution specific level but burn literature describing these patterns is limited.

Prior work on admission variations in burns has used meteorological seasons as markers of variation[14], but to our knowledge, no other study has evaluated burn admission variations on an hour of day basis, and very few have on a day of week basis or day of year basis[15–17]. The goal of this study is to characterize and graphically represent the temporal distribution of burn admissions and analyze how this distribution changes with total body surface area (TBSA) and mechanism. We hypothesize that the distribution of burn admissions falls along predictable temporal patterns and that these distributions change based on TBSA and burn mechanism.

## Methods

Data was acquired from the institutional burn registry, a manually entered database including all burn admissions in a burn center attached to a level-1 trauma center, from July 1, 2016, to March 31, 2021. This study window was based on updates to the electronic medical record that precluded the addition of earlier admissions. This study was conducted as approved by the Vanderbilt Institutional Review Board (IRB Number: 200963). This study is reported in accordance with the STROBE reporting guidelines [18].

The initial cohort included all burn admissions during this period. Exclusion criteria were insufficient recording of admission date, time, TBSA, or burn mechanism. Bivariate absolute and relative frequency data was captured and plotted for all patients who met inclusion criteria. Heat-maps were created to visually represent the relative admission frequency by time of day and day of week. Patients were examined by TBSA and mechanism of injury. TBSA was dichotomized into those at or above 20%, that typically present with severe burn shock requiring acute resuscitation, and those below 20%, that do not [19]. Cohort characteristics are reported with means and standard deviations for normally distributed variables and median with IQR for non-normally distributed variables.

Burn admissions were analyzed both in terms of the absolute number of burns per hour in our center and normalized to the mean number of burns per day. These analyses were repeated for several TBSA groups and burn mechanisms as well. A circular analysis was performed which computed the circular mean hour for each day to compare weekday and weekend admissions. These circular means, which represent the hour of each day where, on average, half of the admissions occurred, were compared using unpaired, 2-tailed t tests. Finally, admissions were plotted by presenting day of the year and normalized by the mean to account for outliers in admission timing. Due to significant variance in burn volume, locally weighted scatterplot smoothing (LOWESS) was used for fitting. We quantified the macro distribution of burn admissions to analyze the temporal pattern over the year. Mathematical, statistical, and graphical analysis was performed with offline MATLAB R2021a (Mathworks, Nantick, MA).

## Results

A total of 2213 non duplicated burn patients were identified in the registry, Table 1. Of these, 131 patients had no data on TBSA, 5 had no admission time, and 2 had neither. These patients were included in the demographics outlined in Table 1 to accurately demonstrate the admission population, but not included in any further analysis, leaving a total of 2075 fully analyzed patients. The mean age of these trauma patients was 47.6 years old (SD: 17.64) and 71.6% were male. The majority were white (85.5%) and the median TBSA was 4.5% (IQR: 1.9 to 9.0%). The most common burn mechanisms were flame and scald burns (58.5% and 22.8%, respectively), with all other mechanisms each individually representing fewer than 10% of all admissions, and when combined less than 20% of all admissions.

**Table 1:** Patient cohort demographics, burn admission characteristics and injury data.

Demographic Characteristics, N = 2213^a^		Count	Percent, SD, or IQR
Male (%)		1571	71%
Mean Age, y (SD)		47.6	17.6
Race (%)			
	White	1892	85.5%
	Black	266	12.0%
	Asian	25	1.1%
	Other Race	18	0.8%
	Unknown	12	0.5%
**Injury Characteristics**			
**TBSA,% (median, IQR; n = 2075** ^**a**^ **)**		4.5	(1.9–9.0)
Burn Mechanism (%)			
	Flame	1295	58.5%
	Scald	504	22.8%
	Contact	133	6.0%
	Chemical	106	4.8%
	Electrical	84	3.8%
	SJS/TEN	45	2.0%
	Electrical	15	0.7%
	Cold Injury	12	0.5%
	Friction	9	0.4%
	Unknown Etiology	5	0.2%
	Electrical	3	0.1%
	Inhalation Injury Only	2	0.1%
**Outcomes**			
	Died (%)	79	3.6%
	Hospital Days (median, IQR)	3	(1–9)

^a^ Demographic data is known for the entire cohort of 2213 patients. TBSA and admission timing were known on 2075 patients, who were included in all subsequent analysis. SD, Standard deviation; IQR, interquartile range; SJS/TEN, Stevens-Johnson syndrome/Toxic epidermal necrolysis.

**Fig 1:**
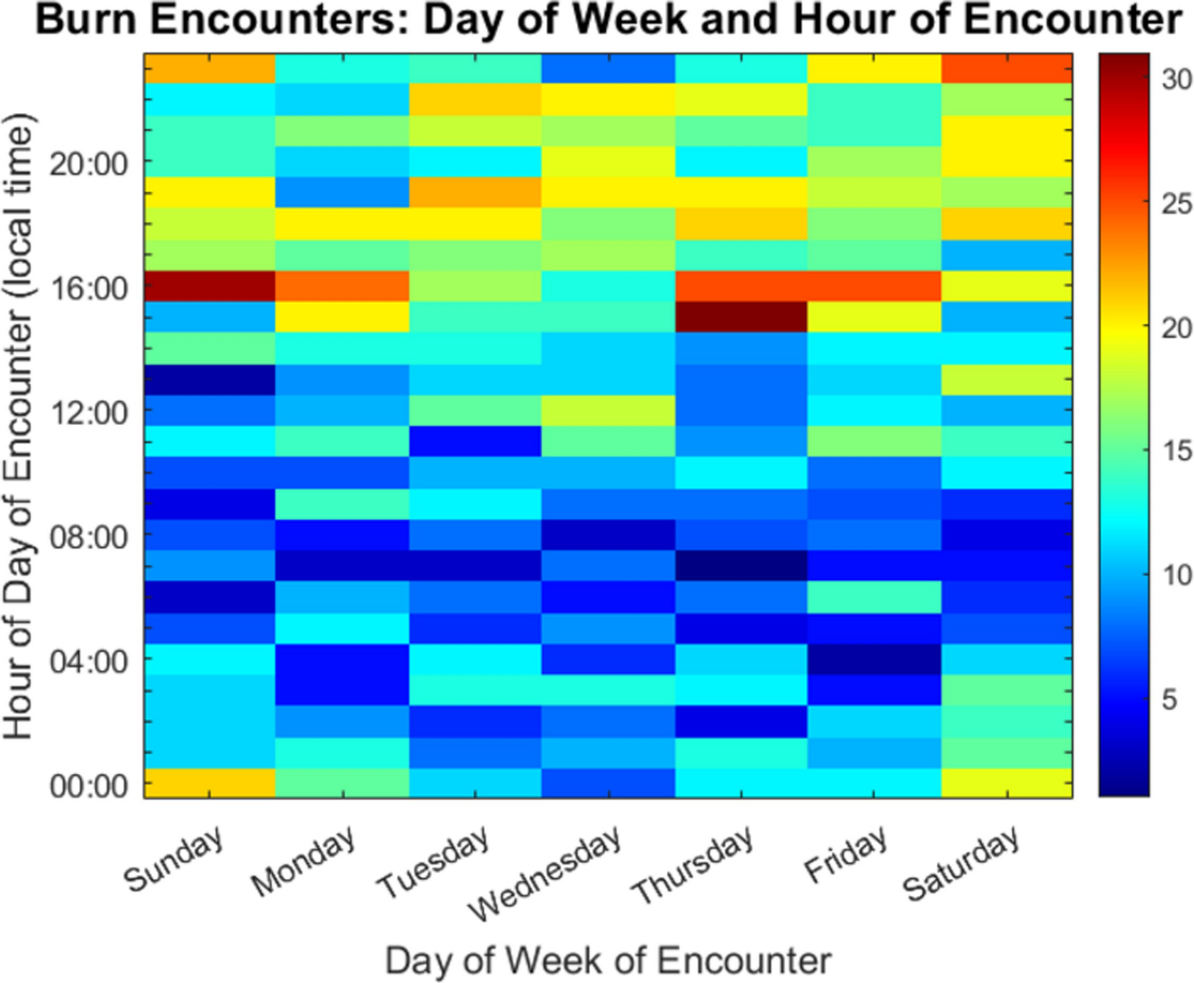
Heatmap of burn admissions by time of day versus day of week. Each block of the heatmap represents the absolute number of admissions in one hour of one day of the week, over the nearly five-year study period. Warm colors represent higher burn admission frequency and cool colors represent lower frequency, as described by the color bar to the right. Late evenings and the early night, (15:00–00:00) have more burn admissions than the rest of the day on all days of the week.

**Fig 2:**
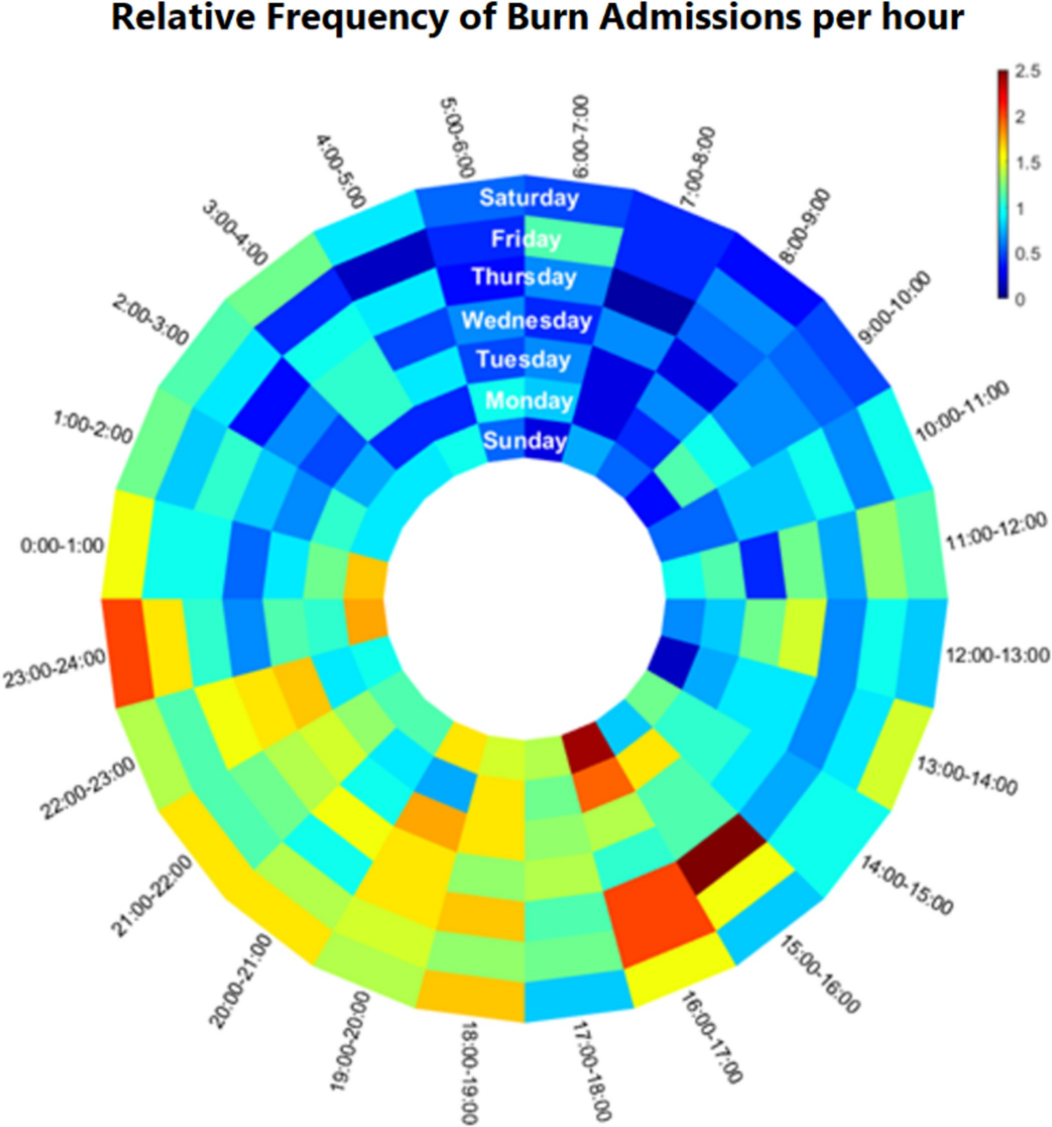
Circular heatmap of relative burn admissions by time of day versus day of week. Each block represents one hour of relative burn admissions normalized to the mean, where 1 represents the mean number of burn admissions per hour. The start of each day was set to the 07:00–08:00 hour, to align with both the timing of clinical handoff and the nadir of daily admissions. On average, half of the weekend daily admissions are done slightly later than the weekday admissions.

The busiest burn admission time was between 15:00 and 00:00; p<0.001. The time of the day which had the fewest burn admissions was between 01:00 and 14:00 (Fig 1). The nadir of admission in all groups was 07:00 hour with a stepwise increase until 16:00, followed by a plateau, and then decreasing admissions starting at 00:00. In terms of absolute admissions, there was only a 53-patient difference in admissions between the highest and lowest admissions days over the entire study period. On initial evaluation, burn activations were not more common on weekends than weekdays, with an average of 15.0% of all burns having presented on each weekend day, vs 14.0% of all burns having presented, on average, each weekday; p = 0.093. Circular analysis weighted for the mean number of burn admissions per hour, per day of the week was performed to further compare weekday admissions to weekend admissions (Fig 2) [20]. The circular mean, corresponding to the hour on each day of the week where half of the admissions had occurred on average, was found to be slightly later on the weekend (20.8, or 20:00 hour) than on the weekday (19.2, or the 19:00 hour); p = 0.025.

**Fig 3:**
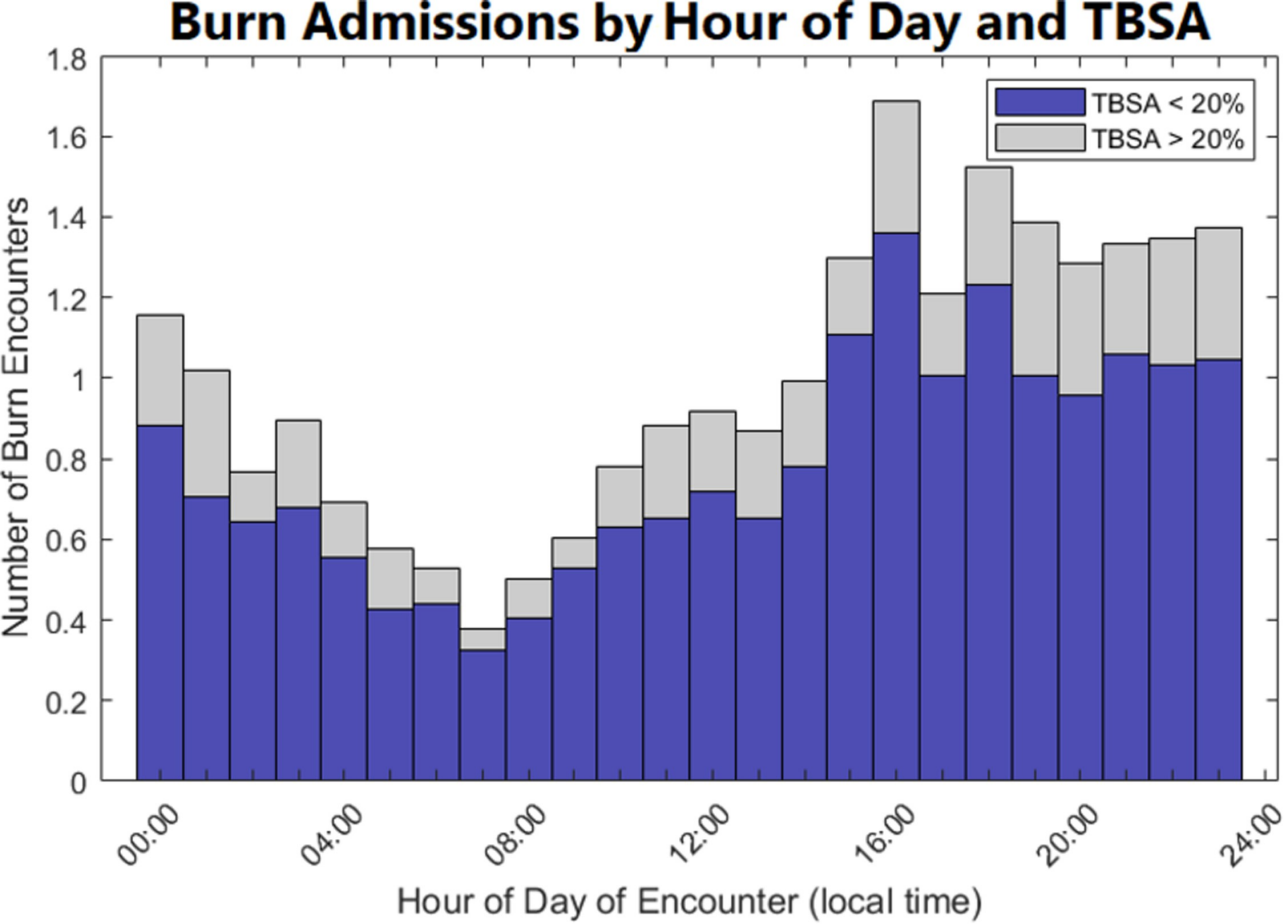
Relative frequency of burn admissions versus time of day partitioned by TBSA<20% and TBSA ≥20%. The height of the bar corresponds to the relative frequency of burn admissions, normalized for the mean number of daily burn admissions. The increase in burn admissions is shown to be primarily driven by burns below 20% TBSA. Larger burns occurred too infrequently to impact the overall pattern of admissions.

**Fig 4:**
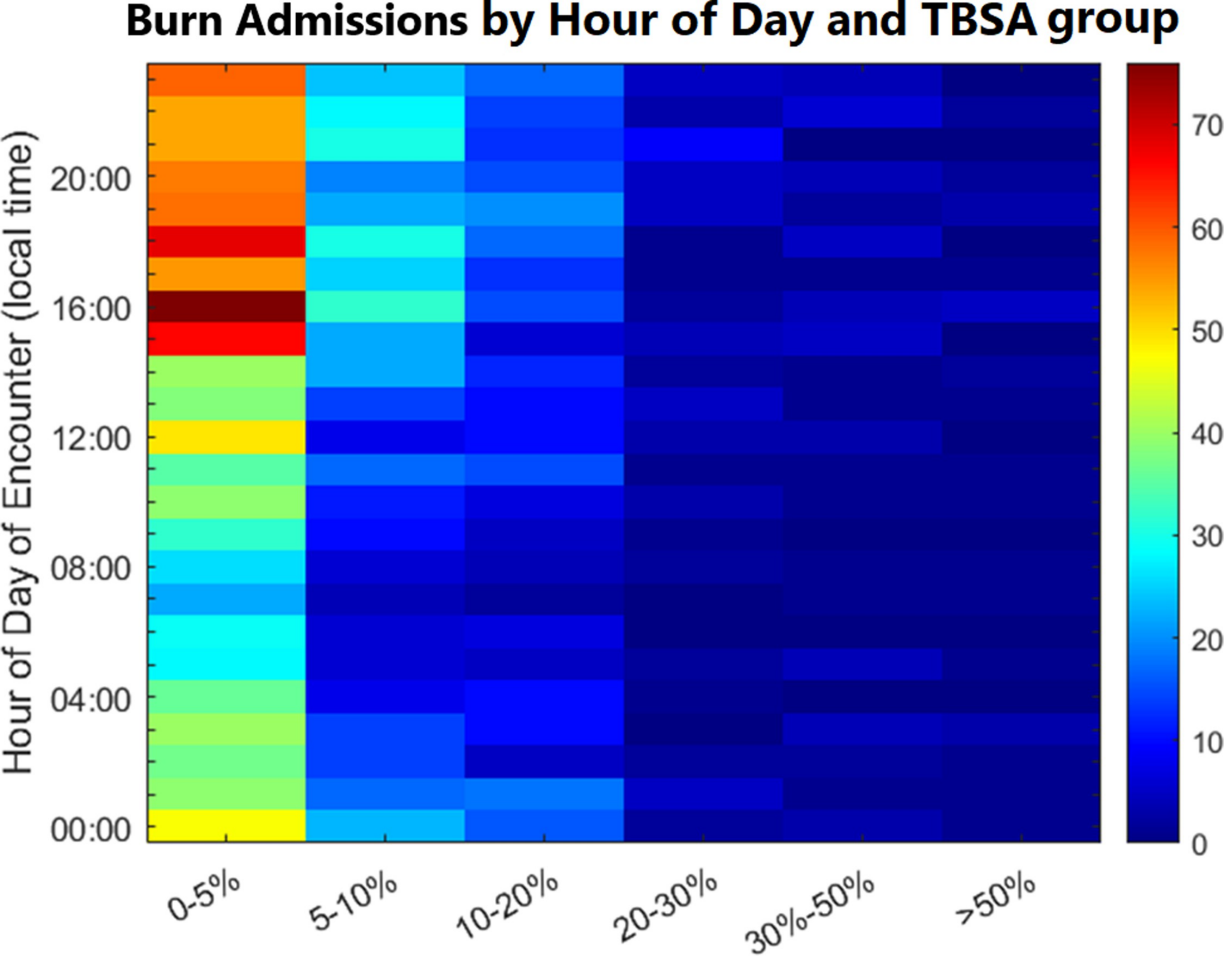
Heatmap of burn admissions by time of day versus total body surface area. The smallest burns, specifically those ≤ 5% TBSA burns account for much of the pattern of burn admissions. The concentration of burn admissions to the late evening and early night (15:00–00:00) is again demonstrated, suggesting that that factors other than TBSA led to the decision to admit patients with burns ≤5%.

When the hourly admission distribution was considered by TBSA group (<20% TBSA vs. ≥20%), burns <20% TBSA were significantly more common and drove the overall pattern (Fig 3). Further stratification by multiple TBSA groups shows that almost all admission time variation was driven by small burns, primarily between 0% and 5% TBSA (Fig 4).

**Fig 5:**
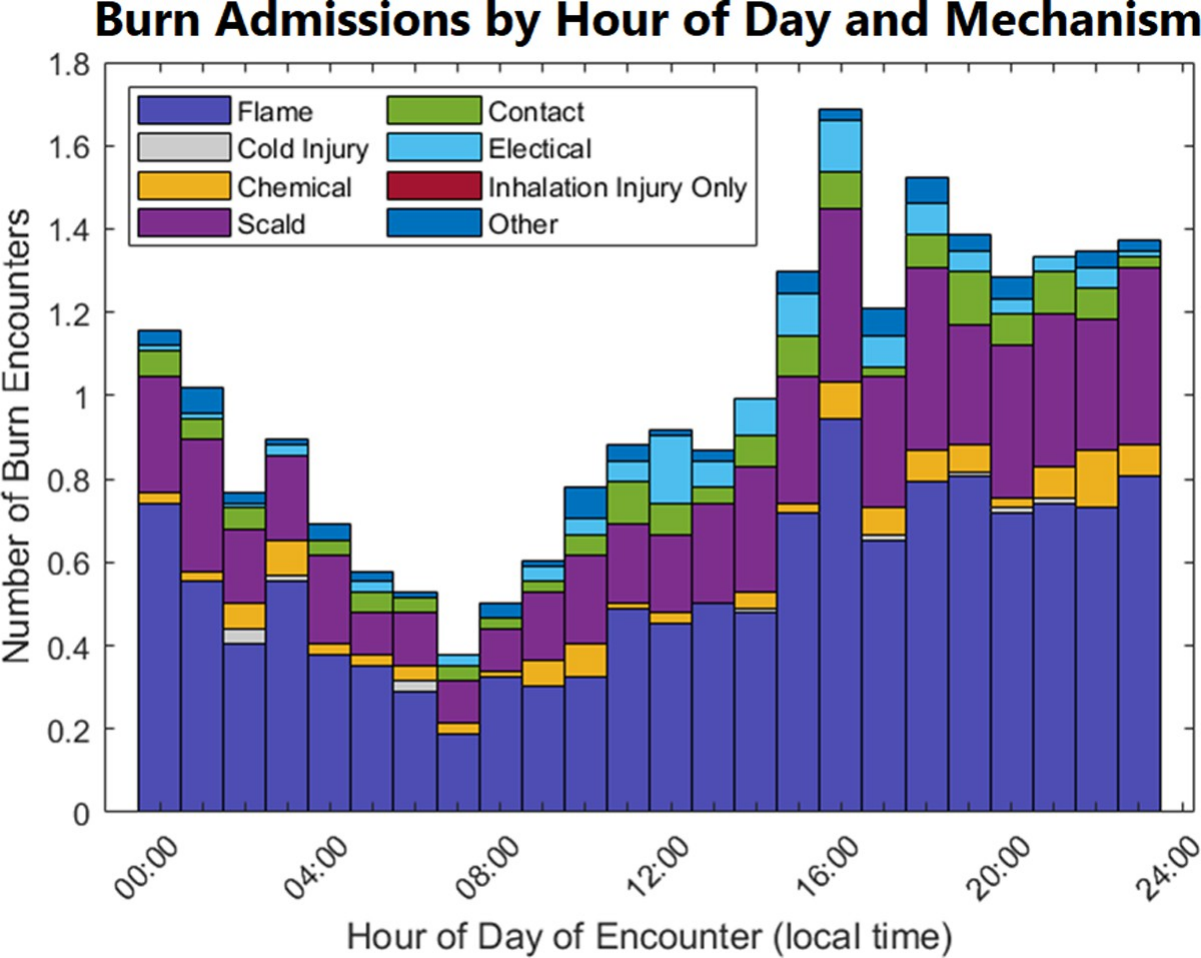
Relative frequency of burn admissions by time of day versus mechanism of injury. The most common burn mechanisms, including flame and scald burns, generate most of the burn admissions and, subsequently, the pattern of burn admission timing. The peak admission window 15:00–00:00) is again demonstrated.

**Fig 6:**
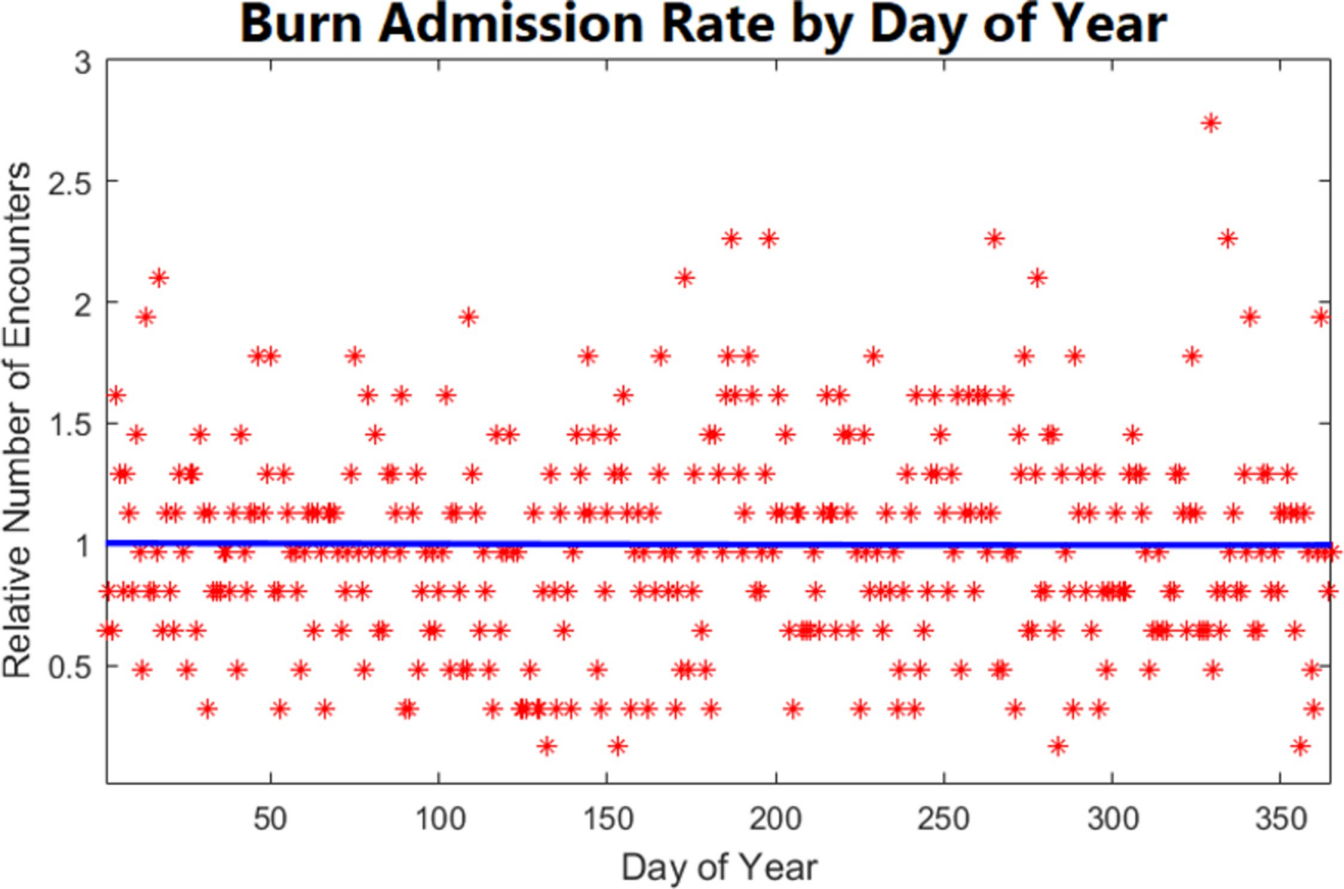
Normalized relative burn frequency per day throughout the year. Relative burn admissions are plotted against the day of the year. The 1 on the y-axis represents the average number of daily burn admissions (1.28 in our data). Each red asterisk represents relative deviations from the mean on that day. A linear regression of those daily variations (blue line) has a slope of 0, indicating that there is no change throughout the year.

The most common mechanisms, specifically flame and scald burns, drove the hourly admission variations, including the daily peaks (Fig 5). We also considered the burn distribution over the year. The asterisks represent daily deviations from the mean number of admissions while the blue line represents the smoothed mean daily burn admissions over the study period (Fig 6). While there were substantial daily variations, the normalized mean frequency of burn admissions versus day of the year did not identify any cyclical trends in admissions.

## Discussion

These data were amalgamated over nearly 5 years and include thousands of admissions. The frequency of admissions rose steadily throughout the day with a peak at 16:00 to 17:00. This peak was maintained on the weekends as well, though quantitatively, the peak and overall high admission volumes, were lower on Sundays and Mondays than on any other days. Sunday evening admissions were qualitatively the nadir of the relative high admission window from 15:00–00:00. These findings are important because 06:00 to 08:00 corresponds to morning handoff and the start of morning rounds in many centers, so an early nadir is protective of that time. However, the evening peak nearly overlaps with evening handoff, which occurs at 18:00. This risks interruptions to handoffs and prolonged in-hospital time for providers. These study findings may be used to re-engineer workflow, such as implementing swing shifts that arrive before peak admission times and stay through them. Alternatively, handoffs could be rescheduled either earlier or later to fit predictable decreases in burn admissions. In our circular analysis, the time by which half of the admissions occurred, on average, was later on the weekends than on the weekdays. In terms of actual timing, however, this difference amounted to either the 20:00 hour or the 19:00 hour, respectively, and likely would not translate into a meaningful change in either staffing or supply decisions.

Hourly admission variations were driven by relatively frequent small burns with common mechanisms, including flame and scald burns. This is in keeping with the relative rarity of burns over 20% TBSA in comparison to smaller burns in the general population [21]. Among burns ≤20%TBSA, the admission time distribution pattern was primarily limited to those ≤5% TBSA, with only minor contribution from the 5–10% TBSA group, and fewer still in the 10–20% TBSA group. At higher TBSAs, the burn admissions were too infrequent to contribute to the overall pattern. Smaller burns were unlikely to require acute burn resuscitation or immediate surgical intervention, but the ones in this data set were admitted, indicating that they had other inpatient needs that were poorly predicted by TBSA alone. The potential inpatient criteria include critical care, pain control, wound care education, or operations. The ability to predict the arrival and admission of patients with small burns requiring admission remains valuable from a resource utilization standpoint, irrespective of TBSA.

Flame and scald burns were common enough to contribute to predictable hourly variation, but do not necessarily correspond to the greatest resource utilization. Less frequent mechanisms, including electrical injuries, chemical injuries, cold thermal injuries, and all other mechanisms, were also too infrequent to affect the admission pattern. However, these less predictable mechanisms represent the most resource intensive and high acuity burn admissions, as they often need decontamination, subspecialty consultation, urgent operative intervention, and an escalation in care driven by factors other than TBSA.

Seen over the span of a year, an average of 1.28 admissions occurred daily. This is a relatively low daily volume from which to identify patterns with certainty but is improved by compiling several years’ worth of data. When the multi-year data are normalized, no predictable seasonal pattern exists in burn admissions (Fig 6). This differs dramatically from previous work in trauma, including our own [22–26], which showed a period from early April to early November that had numbers well above the mean. There are several reasons for this difference. The first is that the known seasonal variations in trauma follow weather patterns conducive to outdoor activities, including motorcycle riding. These seasonal variations do not directly relate to burns. Intuitively, one might think that colder months would lead to more frostbite or to space heater and fireplace use, and other activities that create burn risk, but this is not borne out in the data. Furthermore, we know that flame burns are the primary driver of burn admissions and those can happen from cooking or electrical fires at any time of year.

This study is unique in that it graphically represents burn admissions on an hour-of-day basis, daily, and annual scale in a large burn population. This kind of graphical representation has been shown [27] to help model complex aspects of care to make them more easily understood. Previous studies have identified monthly and daily variations [15] or meteorological seasonal variations [14] in burn admissions. These studies found relative increases on Sundays and Mondays, in July, and in Winter and Summer, but lacked more granular admission data about time of day. In our data, significant hour-of-day burn admissions patterns were identified, but no clear day of week or seasonal variations were found. This is likely due to a difference in admission volumes and length of time evaluated. While ours is also a single-center study, it benefits from amalgamating almost 5 years, rather than extrapolating from a single year’s data. Furthermore, a relatively high burn volume of more than 2000 admissions in less than 5 years has a noise dampening effect on the presence of false patterns in the data analysis.

The limitations of this study include the retrospective design, which limits independent verification or correction of missing or corrupted data fields. It also limits our ability to know the reason for admission, especially those with smaller TBSA. Small TBSA burns were the major contributors to our time-of-day variation, but due to limitations inherent to the retrospective design of this study, the specific admission criteria were not always clear. Our annual data was normalized to the mean number of burn admissions to allow for pragmatic application in other centers with different admission volumes, but this comes with the limitation of unavoidable information loss in a single center study. Smoothing was done to dampen the random variance in day-to-day burn admission numbers, but it also obscured the real impact of events and holidays. We believe this limitation is acceptable, as that was distinctly different from the goals of this study and data on the impact of those events already exists [17, 28–31]. Another limitation is the single center nature of this study. While more than 2000 admissions are modeled over nearly 5 years in this study, local practices may still limit generalizability of the results. As a single center study, it is limited to a specific set of geopolitical and climate patterns. This is the only burn center that services a 50,000 square mile area of the United States, which includes both sparsely populated rural areas and high density- urban areas. However, transfer and admission practices may be unique to this center, beyond broadly accepted ABA guidelines. The region’s climate is temperate and not historically prone to severe cold/icy conditions or wildfires. In other regions, more severe winters or other climate differences may drive admissions differently. Finally, only adults were included in the analysis. Pediatric burn admissions are relatively rare in our center and are divided between the children’s hospital and the adult hospital, effectively doubling the available resources, and making them difficult to include in a pragmatic study meant to guide resource allocation and staffing.

## Conclusion

Using novel graphical and numeric representation, we showed that there is a variation in burn admissions with respect to hour of day, with most admissions occurring in the late afternoon. However, no predictable distribution of burn admissions conforms to either day-of-week or day of year. We showed that small burns (≤ 5% TBSA) and the most frequent burn mechanisms were the primary drivers of the hour of day distribution. These patterns suggest that while the most resource intensive mechanisms and larger burns are less predictable, smaller burns that require admission still tend to occur at predictable times of day and staffing and resources to manage those admissions can be anticipated. In the clinical setting, our findings may be used to guide clinician and support staffing, timing of handoffs, as well as anticipation of interruptions. The ability to predict burn admissions could also be used to guide nurse and aide staffing, bed management, and to avoid overlap in clinical and educational duties for trainees. This study represents the nuances of one burn center, and the geographic and climate context in which it is centered, but this methodology would have to be recreated to reflect the realities of another center.

## Supporting information

S1 Dataset (XLSX)
